# “Cerebellar cognitive reserve”: a possible further area of investigation

**DOI:** 10.1007/s40520-021-01795-1

**Published:** 2021-02-17

**Authors:** Alessandra Bordignon, Maria Devita, Giuseppe Sergi, Alessandra Coin

**Affiliations:** 1 Geriatrics Division, Department of Medicine—DIMED, University of Padua, Via Giustiniani 2, 35128 Padua, Italy; 2 Department of General Psychology—DPG, University of Padua, Via Venezia 12, 35128 Padua, Italy

**Keywords:** Cerebellum, Cerebellar cognitive reserve, Cognitive reserve, Cerebellar atrophy, Dementia

## Introduction

The role of the cerebellum in cognitive functioning has traditionally received little attention, probably because it has always been mainly associated with motor functions. However, clinical and neuroimaging studies have provided convincing evidence that the cerebellum is widely involved in cognition, language and emotions. It has been shown that there is a close network of neural connections with reciprocal feed-forward and feed-back between the cerebellum and areas of the cerebral cortex connected with higher-order behaviors. It is also well-known from the literature that these brain areas may be influenced by specific lifestyles, and the manifestation of clinical symptoms following brain atrophy may be delayed through an appropriate life intervention program. Lived experiences, levels of education and occupation, and mental and physical leisure activities contribute to the formation of the so-called Cognitive Reserve, a series of skills or repertoires which allow some individuals to cope with cognitive decline. Performing cognitively stimulating activities is associated with an increase in brain volume, and cognitive stimulation and exercise can enhance neurogenesis in the dentate gyrus, and increase neuronal plasticity and resistance to cell death. In contrast, a sedentary life with few stimuli may accelerate atrophy of various structures and trigger the early appearance of clinical manifestations. So, what about the cerebellum? Given the analogies and close connections described above, can we speculate that what happens in the brain also happens in the cerebellum? The goal of this point of view is to draw attention to the possible existence of a cerebellar cognitive reserve, which could then be investigated and be the object of intervention. Data in the literature, in fact, show that the cerebellum is an organ that can no longer be underestimated and excluded from consideration of cognitive functions and correlated neurodegenerative processes.

### Brain reserve and cerebellar reserve

The concept of reserve was introduced to describe individual differences in susceptibility to age-related brain changes and pathological changes and has the ability to act as a moderator between pathology and clinical outcomes. Reserve was then differentiated into two categories: brain reserve and cognitive reserve. The concept of brain reserve is quantitative, in that there are, for example, multiple neurons or synapses to lose. It is a passive backup model, as it suggests that the brain can simply tolerate multiple conditions before reaching a critical threshold for the appearance of clinical symptoms. On the other hand, the concept of cognitive reserve suggests that the brain actively tries to cope with brain damage either by using pre-existing cognitive processing strategies or by drawing on compensatory strategies.

According to the literature, the cerebellum is intrinsically capable of self-compensation and restoration, and these abilities are referred to as cerebellar reserve. When acute focal damage occurs (such as in the case of stroke or trauma), the impaired cerebellar function can be compensated for by other cerebellar areas or by extracerebellar structures, and this is termed structural cerebellar reserve. In contrast, when cerebellar neuronal integrity is compromised with consequent gradual cell death (for example in the case of metabolic and immune-mediated cerebellar ataxias or neurodegenerative ataxias), it is possible for the affected area itself to compensate for the cerebellar lesion, and this is termed functional cerebellar reserve [1]. Mitoma and colleagues recently published an interesting work in which they speculate that the cerebellum’s compensatory capacity may be due to its stereotyped and highly geometric architecture, similar to a lattice, and its large number of neurons (60–70% of brain neurons are found in the cerebellum). Two categories of intrinsic mechanisms underlying cerebellar reserve can be derived from this: different types of synaptic plasticity are active, and there is a convergence of central and peripheral information in a given functional unit (microzone) inside the cerebellum and in different microzones, given their modular organization [1].

### Cognitive cerebral reserve and a possible cerebellar cognitive reserve

Cognitive reserve is an active model that describes the flexibility of brain functioning acquired throughout an individual’s lifetime, which can make a person relatively resistant to brain damage before functional deficits emerge. Environmental conditions aimed at improving sensory, motor, cognitive and social stimulation influence not only brain structure and functions but also cerebellar functions [1].

In light of the cerebellum’s architecture and dense neuronal network, is it possible to hypothesize the presence of a cerebellar cognitive reserve that helps compensate for the cerebellar damage described above? And would it also be verifiable through a change in cerebellar volume? Experimental studies show that animal models better compensate for the presence of a cerebellar insult if previously exposed to environmental enrichment [1]. Moreover, morphometric analysis of the cerebellum total volume in humans showed that a group of musicians had significantly larger cerebellar volume than a group of non-musicians [2]. In fact, the cerebellum seems to be particularly important in the early trial-and-error stage of learning motor and cognitive skills, and becomes less active as these skills increase. This suggests the existence of a “cerebellar cognitive reserve” in which, as in the case of cerebral cognitive reserve,^1^ intellectual enrichment is associated with efficiency, which is maintained by greater conservation of activity in the default network/organ resting state and by lesser recruitment of additional regions during cognitive tasks. Furthermore, while it is well known that functional imaging shows cerebellar activation in musicians, such activation has also been observed during the learning of motor sequences and the performance of many non-motor musical skills. It is, therefore, possible that the difference in cerebellar volume observed in musicians is associated not only with a specific motor function but also with cognitive functions, such as motor consolidation or tone recognition [2]. Moreover, other neuroimaging experiments have shown cerebellar task activation and the functional connectivity associated with cognitive control, and have also revealed structural and functional cerebellar anomalies in neurological and psychiatric diseases that impair thought and emotions [1].

### Cerebellum, cerebellar cognitive reserve and dementia

The awareness that most of the human cerebellum is connected to the areas of the brain that influence cognition and the control of emotions defines the revolution that has occurred in our understanding of the cerebellum and the organization of the human brain. Neurodegenerative disorders using the same connectivity network could, therefore, affect both the brain and the cerebellar regions. Neuroimaging and neuromodulation/neurostimulation experiments indicate that cerebellar compensatory reorganization could indeed be relevant even in brain disorders not confined to the cerebellum. Numerous studies have reported differences in cerebellar structure and function in a wide range of neurological and psychiatric disorders that degrade cognition [1]. Interesting results have been obtained, for example, for Alzheimer’s disease, which several studies have found to be associated with lower volumes of cerebellar gray matter and loss of focal volume in specific cerebellar areas. Furthermore, a study has shown cerebellar atrophy to be associated with deficits in various cognitive functions related to specific manifestations of frontotemporal degeneration. The loss of gray matter in lobule VI and in crus I and II is related, for example, to working memory in behavioral variant frontotemporal dementia, as opposed to the visuospatial functions in semantic dementia and motor language skills in progressive non-fluent aphasia patients [3]. Finally, significant associations were found, in patients with Spinocerebellar Ataxia type 2, between atrophy of the posterior lobules of the cerebellar hemisphere and vermis and patients' performance on cognitive tasks involving working memory, phonological fluency, and immediate and delayed recall [4].

The correlation between cerebellum and cognition has led to the acknowledgement of a condition termed cognitive cerebellar affective syndrome (CCAS). It can appear in subjects with restricted cerebellar lesions and is characterized by clinically relevant deficits in executive and visual-spatial functions, a change in personality, and difficulties with language production, all of which could be optimal targets for cognitive stimulation with the aim of improving “cerebellar cognitive reserve”. Finally, recent studies in neuropsychiatry report behavioral improvements in patients with brain disorders after cerebellar neural stimulation [1].

### Possible diagnostic and therapeutic approaches

The topics discussed above raise an interesting question: why is it that while most of the human cerebellum is connected to brain networks important for cognition (with consequent clinical evidence in the case of cerebellar damage/atrophy), clinicians and neuroimaging and neuropsychological researchers nonetheless underestimate and therefore pay little attention to the associated signs and symptoms? To the best of our knowledge, there are currently only one rating scale, no validated in Italy, designed by Hoche and colleagues in 2018 to test cerebellar cognitive capacities; and there is little in the way of a definition, and consequently validation, of cerebellar atrophy scores. Most studies in the literature use complex volumetric analysis techniques, such as voxel-based morphometry, but, in light of the increasing importance the cerebellum is acquiring and its role in cognitive impairment, it would seem there is a need to develop an internationally recognized, easy-to-use score system for evaluating cerebellar atrophy. In a recent study, Carrè and colleagues suggested a possible visual assessment of atrophy performed independently by two physicians (a neurologist and a neuroradiologist) and carried out a brain magnetic resonance imaging analysis of cerebellar atrophy in the T1 weighted sagittal sequence using the following visual scale: 0 = absent, 1 = mild, 2 = moderate, 3 = severe [5]. However, future research in this area is needed. With regard to therapeutic interventions, could cognitive stimulation in healthy elderly people or subjects at symptomatology onset slow cerebellar neurodegeneration with consequent improvement to or stabilization of clinical manifestations? A recent study evaluating 71 measures of cognitive function with 116 patients with cerebellar degeneration or trauma found that Trails Making, Go/No-Go, Category Change, Digit Span Backwards, Verb for Noun Generation, Work Stem Completion, and Phonemic and Semantic Fluency were among the most promising neuropsychological measures. These domains could be optimal targets for exercises to improve cognitive function [1].

## Conclusions

**Fig. 1:**
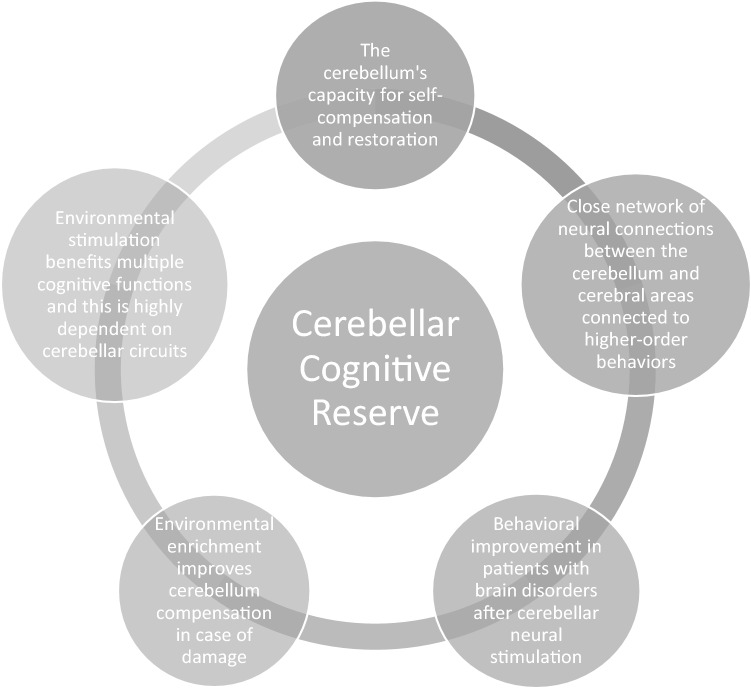
Evidence to support the possible existence of cerebellar cognitive reserve

In light of the evidence reported above, an interesting focus of research appears to be the possible existence of a “cerebellar cognitive reserve”, which could be enhanced to provide protection against cognitive impairment, as already is the case with cerebral cognitive reserve. It has been shown that individuals, even in the presence of brain pathologies, are able to adapt effectively to environmental needs, and exposure to complex environmental stimulation benefits multiple cognitive functions. The capacity for cognitive adaptation is highly dependent on cerebellar circuits and seems to exploit two cerebellar neuronal properties: multiform synaptic plasticity and redundant information processing [1]. Motor and cognitive rehabilitation and non-invasive cerebellar stimulation with the aim of modulating these properties could represent effective therapeutic treatments in cases of cerebellar lesion and also cerebral pathologies. Further in-depth research is needed in this area (Fig. 1).

## Footnotes

1 The term “cerebral cognitive reserve” is analogous to the definition, well-known in the literature, of Cognitive Reserve, to differentiate it from our hypothesis regarding the existence of a purely “cerebellar cognitive reserve”.
